# Incidence of obesity and its predictors in children and adolescents in 10 years of follow up: Tehran lipid and glucose study (TLGS)

**DOI:** 10.1186/s12887-018-1224-6

**Published:** 2018-07-25

**Authors:** Maryam Barzin, Shayan Aryannezhad, Sara Serahati, Akram Beikyazdi, Fereidoun Azizi, Majid Valizadeh, Maryam Ziadlou, Farhad Hosseinpanah

**Affiliations:** 1 Obesity Research Center, Research Institute for Endocrine Sciences, Shahid Beheshti University of Medical Sciences, Tehran, Iran; 2 Endocrine Research Center, Research Institute for Endocrine Sciences, Shahid Beheshti University of Medical Sciences, Tehran, Iran

**Keywords:** Obesity, Childhood, Adolescents, Incidence, Predictors

## Abstract

**Background:**

Childhood obesity is one of the most challenging public health issues of twenty-first century. While we know that there is an increase in prevalence of childhood and adolescence obesity, incidence studies must be carried out. The main objective of this study was to determine childhood obesity incidence and its potential predictors in Tehranian urban population.

**Methods:**

This study was conducted within the framework of the Tehran Lipid and Glucose Study (TLGS), addressing incidence and risk factors of obesity throughout several phases from 1999–2001 to 2009–2011 among Tehranian urban population. Total study subjects were 1033 non-obese children, aged between 7 to 11 years, with a median 8.7 years of follow-up. Body mass Index (BMI) was used to define obesity and overweight based on World Health Organization (WHO) criteria, and definition of metabolic syndrome (MetS) for children was based on the Cook survey. Cumulative incidence of obesity and obesity incidence rates were calculated for each gender. Cox proportional hazard models was used to estimate potential risk factors of obesity.

**Results:**

Our Participants had a mean age of 9.2 ± 1.4 years, mean BMI of 16.1 ± 2.2 kg/m^2^ and mean waist circumference (WC) of 57.2 ± 6.7 at baseline. Total cumulative incidence of obesity was calculated to be 17%, CI =14.1–20.4 for whole population (19.6%, CI =15.4–24.8 for boys and 14.5%,CI = 10.9–19.1 for girls). Participants which were in the age group of 7–9 years at baseline experienced higher rate of cumulative obesity incidence compared to those who were in the age group of 10–11 years at baseline (22% vs 10.8%).

In addressing risk factors, 5 parameters were significantly associated with obesity incidence: being overweight at baseline (HR = 14.93 95%CI: 9.82–22.70), having higher WC (HR = 5.05 95%CI: 3.01–8.48), suffering from childhood MetS (HR: 2.77 95%CI: 1.57–4.89) and having a obese father (HR: 2.69 95%CI: 1.61–4.50) or mother (HR: 3.04 95%CI: 1.96–4.72).

**Conclusion:**

Incidence of obesity is significantly high in Tehranian children, especially the age group 7–9 years. Best predictors of childhood obesity incidence are childhood overweight, WC above 90th percentile, childhood MetS and parental obesity.

## Background

Based on World Health Organization (WHO) reports, childhood obesity is one of the most serious global health challenges of the twenty-first century which is steadily affecting many low- and middle-income countries [1]. It has also been stated that overweight or obese children are more likely to remain overweight or obese in adulthood [2]. This persistency of obesity into the adulthood is associated with increased morbidity risk in later life, leading to development of adult diabetes, coronary heart disease and a range of cancers [3].

Increase in the prevalence of overweight and obesity has been detected among children and adolescents worldwide, making obesity one of the most common chronic disorders in this age group [4]. In a 2017 systematic review; global and regional prevalence of obesity among 5–19 years old children and adolescents was published. The study showed an increasing trend of obesity worldwide; prevalence of obesity in 1975 was 0.7% in girls and 0.9% in boys, rising to 5.6% in girls and 7.8% in boys in 2016. The Middle East and north Africa (MENA) region was among the regions with the largest absolute increase in the number of children and adolescents with obesity globally (around or above 20%, in some countries). These findings highlight the growing concern of the rising prevalence of childhood overweight and obesity in this region [5].

A national based study in Iran, a developing country in Middle East region, showed prevalence of overweight and obesity among children and adolescents to be high, 14.5 and 6% respectively [6]. Estimations of childhood and adolescence obesity prevalence reported by Tehran Lipid and Glucose Study (TLGS) are comparable with this previously published national based study: prevalence of overweight and obesity, were demonstrated be 13.3 and 4.3% respectivly (for 3–19 years old) in TLGS population at phase I of study (1999–2001) [7].

Findings of a recent systematic review and meta-analysis study of Iranian children and adolescents revealed an alarming increase in the trend of excess weight in children aged below 11 years compared with older children. [8]. Although prevalence of childhood obesity has been reported in many studies, incidence studies are needed to determine potential risk factors for developing obesity. Despite its importance, there is limited knowledge regarding childhood obesity incidence. Studies carried out based on nationally representative data in the U.S and England, investigated different childhood ages to ascertain as the most probable for incidence of obesity and the results are rather conflicting [9, 10], with the effects of different risk factors on the age of obesity incidence still being under question.

This longitudinal population based cohort study aimed to determine childhood obesity incidence in a Tehranian urban population, and to evaluate the potential predictors of obesity incidence in this sample.

## Methods

### Study setting and participants

This prospective study was conducted within the framework of the Tehran Lipid and Glucose Study (TLGS), a population based cohort study aimed at determining the risk factors of non-communicable diseases among Tehranian population. Details of this study protocol are available elsewhere [11]. Tehran, the capital of the Islamic Republic of Iran, is a metropolitan city composed of 22 urban districts, which make up a population of more than 8.6 million people (based on Iran National Census 2016). All participants were chosen from the urban District 13 of Tehran via multistage cluster random sampling method and were given a written invitation form. Rational for choosing district 13 as a representative of the overall population of Tehran is its high stability of the residing population and its age distribution which is similar to whole Tehran. Based on the written data, every family was contacted, invited, and then recruited to participate in the study and was referd to one of the three chosen medical health centers in district 13 for the measurements and next follow-ups. TLGS consists of several phases, phase I (1999–2001), a cross-sectional prevalence study of cardiovascular risk factors, in which, 15,005 people, aged ≥3 years were selected; then a prospective follow up study was conducted with phases II (2002–2005), III (2006–2008) and IV (2009–2011) by means of approximately 3 years intervals between assessments. Moreover, during phase II, 3500 new participants were recruited.

This study has been approved by the National Research Council of the Islamic Republic of Iran (No. 121) and has been performed with the approval of the Human Research Review Committee of the Endocrine Research Center, Shahid Beheshti University (M. C).

**Fig. 1:**
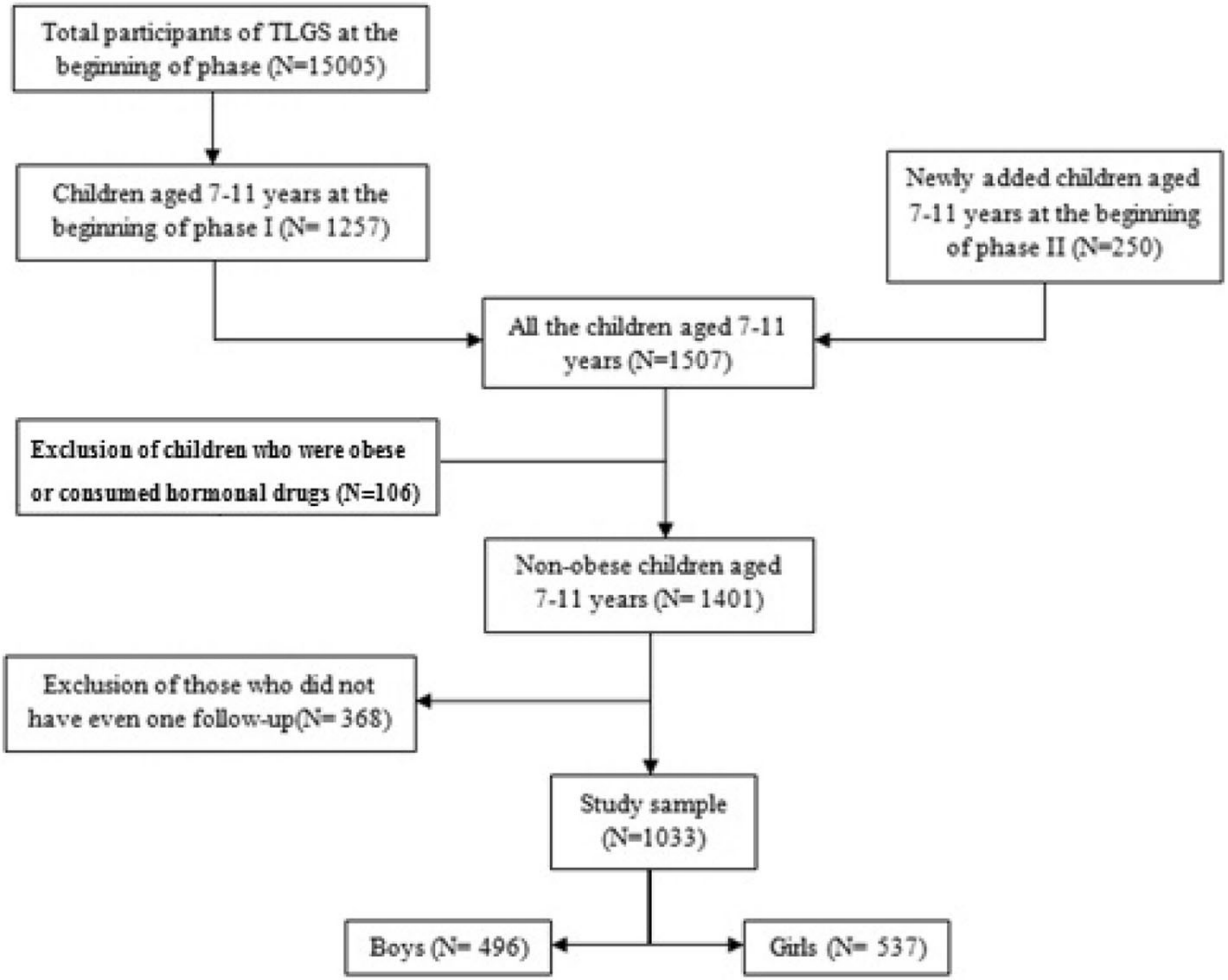
Flow chart of inclusion and exclusions of study participants

In the current study, participants aged between 7 to 11 years entered study at first 2 phases, total 1507 participants from phase I (*N* = 1257) and II (*N* = 250) were selected. After exclusion of those who were obese at baseline and those with consumption of glucocorticoids or other hormonal drugs (total number of exclusions *N* = 106); 1401 participants remained. Of these participants, 368 had no further follow-up. Final analysis were performed on 1033 participants for a median of 8.7 years [dropout rate about 26.3% (368 of 1401)] (Fig. 1).

### Measurements and definitions

Trained interviewers collected information regarding demographics, education, medical and drug history. All measurements were taken by trained technicians in order to reduce subjective errors.

Anthropometric parameters: Weight was measured according to standard protocols with an accuracy of up to 100 g, with subjects minimally clothed without shoes using digital scales. Height was measured in a standing position, without shoes, using a tape measure while the shoulders were in a normal position. BMI was calculated as weight in kilograms divided by the height in squared meters (kg/m^2^). Waist circumference (WC) was measured at the narrowest level over light clothing, using an un-stretched tape meter, without any pressure to body surface, and measurements were recorded to the nearest 0.1 cm. Based on BMI-for-age standards of WHO, obesity for children was defined as BMI-for-age > 2SD and overweight was defined as 1SD < BMI-for-age ≤ 2SD in each gender [12]; parental obesity was defined as BMI ≥30.

Blood pressure and metabolic parameters: A qualified physician, using a standard mercury sphygmomanometer, measured systolic and diastolic blood pressure two times on the right arm, with the subject in a seated position, asked to rest for 15 min period between measurements. The mean of two measurements was considered to be the participant’s blood pressure. Blood samples were drawn from all the study participants after an overnight fasting of 12–14 h. All blood analyses were performed at the TLGS research laboratory on the day of blood collection. Fasting plasma glucose (FPG) was measured by the enzymatic colorimetric method using glucose oxidase. Plasma total cholesterol (TC) and triglyceride (TG) levels were measured by enzymatic colorimetric kits using cholesterol esterase/cholesterol oxidase and glycerol phosphate oxidase respectively. High-density lipoprotein cholesterol (HDL-C) was measured after precipitation of the apolipoprotein B–containing lipoproteins with phosphotungstic acid. Definition of metabolic syndrome (MetS) for children was based on the Cook et al. survey [13]. This definition is based on criteria analogous to that of the National Cholesterol Education Program Expert Panel on Detection, Evaluation and Treatment of High Blood Cholesterol in Adult Treatment Panel III [14] and it defines MetS as three or more of the following: Fasting TG ≥ 110 mg/dl; HDL cholesterol < 40 mg/dl; WC ≥ 90th percentile for age and gender, according to national reference curves [15]; systolic blood pressure (SBP) and/or diastolic blood pressure (DBP) ≥90th percentile for gender, age and height according to Heart, Lung, and Blood Institute standards and FPG ≥ 100 mg/dl [16].

Education: Parental educational levels were assessed using a questionnaire and were categorized into two groups, >high school diploma and ≤ high school diploma. In Iran, it took 12 years of education to receive a high school diploma.

### Statistical analysis

Normality of distributions was checked using the Kolmogorov-Smirnov test for all continuous variables. Normally distributed and skewed continuous variables are illustrated as mean ± SD and median (IQ 25–75), respectively. Categorical variables are reported as frequency (percentages). To assess the significance of differences for categorical and continuous variables in the baseline characteristics of all participants at follow-up, Pearson chi square test, t-test and Mann-Whitney test were used, when appropriate.

In this study, as the exact time of obesity incidence was not known, it was considered as interval-censored data. Considering alternate interval censoring approaches, results were investigated using mid-point censoring, which converts interval-censored data to the right-censored data problems. Mid-point censoring was set to the mid-point between the last negative and the most recent positive event time minus the first positive observation for the incidence of obesity and to the time span between the first and the last observation for censored subjects. End points were considered as the time of incidence of obesity and censoring was defined as lost to follow up or end of the follow up.

Cumulative incidence of obesity with 95% (CI) was calculated for each gender as the number of new cases of obesity over the total number of subjects in that group minus half of the censored population. The person-year method was used to obtain obesity incidence rates (IRs); IR is reported as number of cases per 1000 person years. Cox proportional hazard modeling was used to estimate unadjusted and age adjusted hazard ratios (HRs) along with 95% (CI) for baseline components of MetS, parental obesity and educational level. The proportionality assumption was verified by assessing the correlation between the Schoenfield residuals and person-days along with observing log minus log plots (considering different groups as strata variables). All proportionality assumptions were generally appropriate. All analyses were performed using IBM SPSS for Windows version 20 and STATA version 12 SE (STATA Inc., TX, USA), with a two-tailed *P*-value, 0.05 being considered significant.

## Results

Total of 1033 non-obese participants (496 males, 537 females) with a mean age of 9.2 ± 1.4 years, mean BMI 16.1 ± 2.2 kg/m^2^ and mean WC 57.2 ± 6.7 cm at baseline were followed up for a median of 8.7 (IQ = 5.5–10.4) years. Prevalence of overweight was 14.9% (*n* = 154) at baseline, 16.3% (*n* = 81) in boys and 13.6% (*n* = 73) in girls. Baseline characteristics of the study participants separated by gender are shown in Table 1 indicating a non-significant difference between different genders in their demographic and biochemical characteristics except for WC, mother’s BMI, FPG, TG and SBP.

**Table 1:** Baseline characteristics of study participants

Characteristic	Boys (*N* = 496)	Girls (*N* = 537)	Total (*N* = 1033)	*P*-value^b^
Age (years)	9.1 ± 1.5	9.2 ± 1.4	9.2 ± 1.4	NS
BMI (kg/m^2^)	16.1 ± 2.0	16.0 ± 2.3	16.1 ± 2.2	NS
Overweight n(%)	81 (16.3)	73 (13.6)	154 (14.9)	*< 0.05*
WC (cm)	56.4 ± 6.0	57.9 ± 7.1	57.2 ± 6.7	*< 0.05*
WC ≥90th (cm) n(%)	32 (6.5)	32 (6.0)	64 (6.2)	NS
FPG (mg/dl)	88.2 ± 8.8	86.4 ± 7.7	87.3 ± 8.3	*< 0.05*
FPG ≥ 100 (mg/dl) n(%)	44 (9.3)	24 (4.6)	69 (6.9)	*< 0.05*
HDL-C (mg/dl)	47.6 ± 11.4	46.3 ± 11.2	46.9 ± 11.3	NS
HDL-C < 40 (mg/dl) n(%)	126 (27.0)	161 (31.1)	287 (29.2)	NS
TG^a^ (mg/dl)	80.0 (61.0–103.0)	88.0 (70.0–118.0)	84.0 (64.0–111.2)	*< 0.05*
TG ≥ 110 (mg/dl) n(%)	101 (21.5)	160 (30.7)	261 (26.4)	*< 0.05*
Paternal BMI (kg/m^2^)	26.4 ± 3.6	25.7 ± 3.7	26.0 ± 3.8	NS
Paternal obesity n(%)	51 (14.5)	52 (13.6)	103 (14.0)	NS
Maternal BMI (kg/m^2^)	27.7 ± 4.8	27.6 ± 3.0	27.5 ± 4.6	*< 0.05*
Maternal obesity n(%)	120 (28.2)	113 (24.1)	233 (26.1)	NS
Paternal educational level				
Higher than diploma n(%)	71 (18.0)	67 (16.1)	138 (17.5)	NS
Maternal educational level				
Higher than diploma n(%)	55 (11.7)	48 (9.5)	103 (10.5)	NS
MetS n(%)	26 (5.5)	40 (7.7)	66 (6.7)	NS
SBP (mmHg)	102.7 ± 11.6	100.2 ± 12.1	101.3 ± 11.9	*< 0.05*
DBP(mmHg)	70.2 ± 10.2	69.3 ± 10.2	69.8 ± 10.2	NS
Hypertension n(%)	163 (33.6)	180 (33.8)	343 (33.7)	NS

*BMI* body mass index, Overweight, 1SD < BMI-for-age ≤ 2SD based on WHO criteria; *WC* waist circumference, *FPG* fasting plasma glucose, *HDL-C* high-density lipoprotein cholesterol, *TG* triglycerides, Father’s obesity, Father’s BMI ≥ 30 kg/m^2^; Mother’s obesity, Mother’s BMI ≥ 30 kg/m^2^; *SBP* systolic blood pressure, *DBP* diastolic blood pressure, Hypertension, SBP and/or DBP ≥90th percentile for gender and age

^a^median IQ 25–75

^b^between genders differences

**Fig. 2:**
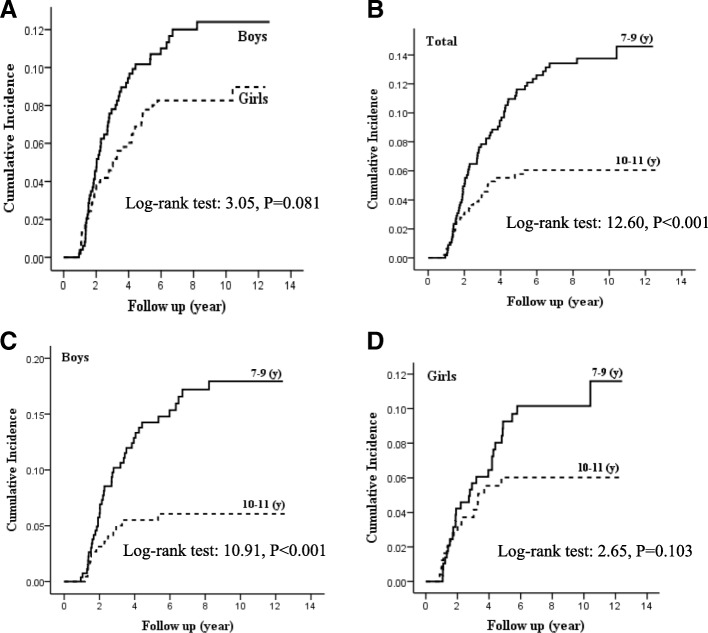
Kaplan-Meier Curve for cumulative incidence of obesity; **a** Stratified by gender, **b** Stratified by different age groups, **c** Stratified by different age groups of boys, **d** Stratified by different age groups of girls

At the end of follow up, 4.0% (*n* = 35) of the normal weight subjects and 39.6% (*n* = 61) of overweight subjects at baseline, became obese contributing to a cumulative incidence of 17.0% (CI 14.1–20.4%). Gender striated cumulative incidence was 19.6% (CI 15.4–24.8%) and 14.5% (CI 10.9–19.1%) for boys and girls, respectively. For the whole population, incidence density rate was 11.8 (9.7–14.4) per 1000 person year, and corresponding incidence density rates among boys and girls were 14.3 (10.9–18.7) and 9.7 (7.2–13.1) per 1000 person year, respectively. Kaplan-Meier curve (Fig. 2a) shows that boys are at increased risk of obesity, compared to girls, also it is not statistically significant. (Log-rank test: 3.05 *P* = 0.081). As shown in Table 2, contributions of different candidate predictor of incidence of obesity were analyzed and corresponding HRs were calculated for the whole population. Once the adjustments for baseline age were performed, Being overweight and having WC of ≥90th percentile at baseline had significant association with incidence of obesity; (HR = 14.93 95%CI:9.82–22.70) and HR = 5.05 95%CI: 3.01–8.48) respectively. Childhood MetS (HR: 2.77 95% CI: 1.57–4.89) and parental obesity (HR: 2. 69 95% CI: 1.61–4.50 and HR: 3.04 95% CI: 1.96–4.72 for paternal and maternal obesity, respectively), also had a significant association with incidence of obesity. However, other parameters including HDL cholesterol, hypertension, fasting blood sugar and parental educational levels showed no significant association with developing obesity in the whole population. After separating data by gender (Tables 3 and 4) the same pattern was observed for all the covariates except for paternal obesity in girls which had a non-significant association with obesity incidence.

**Table 2:** Hazard ratios and 95% confidence intervals of potential risk factors in whole population

Variables	Cumulative Incidence of Obesity (95% CI)	Incidence rate (in 1000 person year)	Un-adjusted HR (95% CI)	Adjusted HR^a^ (95% CI)
Total	17.0 (14.1–20.4)	11.8 (9.7–14.4)	–	
Gender				
Boys	19.6 (15.4–24.8)	14.3 (10.9–18.7)	1	1
Girls	14.5 (10.9–19.1)	9.7 (7.2–13.1)	0.70 (0.47–1.05)	0.72 (0.48–1.07)
Weight groups				
Normal weight	7.7 (5.6–10.5)	4.8 (3.5–6.7)	1	1
Overweight	56.7 (47.6–66.2)	68.8 (53.5–88.4)	13.48 (8.88–20.46)	14.93 (9.82–22.70)
WC ≥ 90th				
No	14.9 (12.1–18.3)	10.1 (8.1–12.6)	1	1
Yes	43.9 (30.4–60.3)	50.3 (32.7–79.8)	4.49 (2.68–7.50)	5.05 (3.01–8.48)
FPG ≥ 100 (mg/dl)				
No	16.8 (13.8–20.4)	11.7 (9.5–14.5)	1	1
Yes	18.7 (9.4–35.2)	14.7 (6.5–28.8)	1.13 (0.52–2.44)	1.25 (0.57–2.69)
TG ≥ 110 (mg/dl)				
No	16.6 (13.3–20.6)	11.6 (9.1–14.7)	1	1
Yes	17.5 (12.2–24.8)	12.1 (8.2–17.9)	1.07 (0.68–1.70)	1.17 (0.74–1.86)
HDL-C < 40 (mg/dl)				
No	16.1 (12.8–20.2)	11.2 (8.7–14.4)	1	1
Yes	18.9 (13.6–25.9)	13.3 (9.3–19.1)	1.21 (0.78–1.87)	1.20 (0.77–1.86)
Hypertension				
No	14.8 (11.6–18.9)	10.4 (7.9–13.5)	1	1
Yes	21.8 (16.6–28.3)	15.2 (11.2–20.6)	1.53 (1.02–2.29)	1.35 (0.90–2.03)
MetS				
No	15.5 (12.6–19.0)	10.6 (8.5–13.3)	1	1
Yes	35.0(22.4–51.8)	31.28 (18.0–52.8)	2.84 (1.61–5.02)	2.77 (1.57–4.89)
Paternal obesity				
Non-obese	14.1 (10.8–18.3)	9.1 (6.9–12.1)	1	1
Obese	33.9 (23.6–47.1)	26.8 (17.5–41.1)	2.83 (1.70–4.72)	2.69 (1.61–4.50)
Maternal obesity				
Non-obese	12.2 (9.2–16.1)	7.7 (5.7–10.4)	1	1
Obese	28.0 (21.3–36.4)	21.6 (15.7–29.7)	2.69 (1.74–4.16)	3.04 (1.96–4.72)
Paternal educational level				
Diploma or lower than diploma	17.1 (13.6–21.4)	11.9 (9.3–15.3)	1	1
Higher than diploma	18.4 (11.3–29.1)	12.7 (7.5–21.5)	1.07 (0.60–1.90)	1.11 (0.62–1.99)
Maternal educational level				
Diploma or lower than diploma	17.1 (14.0–20.8)	11.7 (9.4–14.5)	1	1
Higher than diploma	19.3 (11.2–32.1)	16.3 (9.1–29.5)	1.25 (0.67–2.35)	1.20 (0.64–2.26)

Overweight, 1SD < BMI-for-age ≤ 2SD based on WHO criteria; *WC* waist circumference; *FPG* fasting plasma glucose; HDL-C, high-density lipoprotein cholesterol; *TG* triglycerides; Paternal obesity, Father’s BMI ≥ 30 kg/m^2^; Maternal obesity, Mother’s BMI ≥ 30 kg/m^2^; Hypertension, SBP and/or DBP ≥90th percentile for gender and age; MetS, Metabolic syndrome for children based on the definition of Cook et al. work

^a^Adjusted for age at baseline

**Table 3:** Hazard ratios and 95% confidence intervals of potential risk factors in boys

Variables	Cumulative Incidence of Obesity (95% CI)	Incidence rate (in 1000 person year)	Un-adjusted HR (95% CI)	Adjusted HR^a^ (95% CI)
Weight groups				
Normal weight	10.5 (7.1–15.3)	6.9 (4.6–10.4)	1	1
Overweight	55.4 (43-68.6)	66.8 (47–94.9)	9.16 (5.33–15.47)	10.42 (6.04–17.97)
WC ≥ 90th				
No	16.6 (12.6–21.8)	11.6 (8.5–15.6)	1	1
Yes	54.6 (35.7–75.6)	82.3 (46.8–145.0)	5.93 (3.11–11.32)	9.31 (4.62–18.73)
FPG ≥ 100 (mg/dl)				
No	19.0 (14.6–24.7)	13.8 (10.3–18.4)	1	1
Yes	20.4 (9.0–42.2)	16.0 (6.7–38.5)	1.10 (0.44–2.77)	1.32 (0.45–2.85)
TG ≥ 110 (mg/dl)				
No	20.0 (15.1–26.2)	14.8 (10.9–20.2)	1	1
Yes	14.7 (7.6–27.2)	9.9 (5.0–19.8)	0.70 (0.32–1.48)	0.72 (0.34–1.54)
HDL-C < 40 (mg/dl)				
No	17.7 (12.9–24.0)	12.8 (9.0–18.0)	1	1
Yes	22.5 (14.5–34.1)	16.7 (10.2–27.3)	1.32 (0.73–2.40)	1.18 (0.65–2.15)
Hypertension				
No	18.0 (13.1–24.6)	13.2 (9.3–18.7)	1	1
Yes	23.8 (16.3–33.8)	17.4 (11.5–26.5)	1.37 (0.80–2.36)	1.24 (0.72–2.14)
MetS				
No	18.0 (13.7–23.4)	12.9 (9.6–17.4)	1	1
Yes	37.5 (18.9–65.1)	32.5 (15.8–87.4)	2.58 (1.10–6.06)	2.33 (1.00–5.48)
Paternal obesity				
Non-obese	14.8 (10.1–21.3)	9.8 (6.5–14.6)	1	1
Obese	47.8 (32.6–65.7)	46.5 (28.5–76.0)	4.41 (2.34–8.30)	3.93 (2.08–7.42)
Maternal obesity				
Non-obese	15.1 (10.5–21.5)	10.0 (6.7–14.8)	1	1
Obese	26.0 (17.3–38.1)	19.8 (12.5–31.5)	1.92 (1.05–3.52)	2.14 (1.66–3.94)
Paternal educational level				
Diploma or lower than diploma	19.6 (14.4–26.1)	14.2 (10.2–19.8)	1	1
Higher than diploma	22.5 (12.4–38.8)	17.0 (8.8–32.6)	1.16 (0.56–2.41)	1.15 (0.55–2.34)
Maternal educational level				
Diploma or lower than diploma	20.2 (15.6–26.0)	14.4 (10.9–19.2)	1	1
Higher than diploma	16.7 (7.3–35.5)	14.3 (6.0–34.4)	0.87 (0.35–2.19)	0.68 (0.55–0.83)

Overweight, 1SD < BMI-for-age ≤ 2SD based on WHO criteria; *WC* waist circumference, *FPG* fasting plasma glucose, *HDL-C* high-density lipoprotein cholesterol, *TG* triglycerides, Paternal obesity, Father’s BMI ≥ 30 kg/m^2^; Maternal obesity, Mother’s BMI ≥ 30 kg/m^2^; Hypertension, SBP and/or DBP ≥90th percentile for gender and age; MetS, Metabolic syndrome for children based on the definition of Cook et al. work

^a^Adjusted for age at baseline

**Table 4:** Hazard ratios and 95% confidence intervals of potential risk factors in girls

Variables	Cumulative Incidence of Obesity (95% CI)	Incidence rate (in 1000 person year)	Un-adjusted HR (95% CI)	Adjusted HR^a^ (95% CI)
Weight groups				
Normal weight	5.0 (2.9–8.7)	3.0 (1.7–5.4)	1	1
Overweight	58.2 (45.3–71.7)	71.0 (49.7–101.5)	21.8 (11.14–42.67)	23.42 (11.93–45.96)
WC ≥ 90th				
No	13.3 (9.8–18.0)	8.7 (6.3–12.1)	1	1
Yes	31.6 (15.6–57.2)	28.3 (12.7–62.9)	3.09 (1.30–7.33)	3.12 (1.31–7.41)
FPG ≥ 100 (mg/dl)				
No	14.9 (11.2–19.8)	10.0 (7.3–13.6)	1	1
Yes	10.0 (4.1–48.8)	10.1 (2.5–40.4)	1.01 (0.21–4.19)	1.23 (0.29–5.14)
TG ≥ 110 (mg/dl)				
No	13.0 (9.0–18.6)	8.5 (5.8–12.6)	1	1
Yes	19.2 (12.4–29.1)	13.5 (8.4–21.7)	1.60 (0.86–2.96)	1.77 (0.95–3.29)
HDL-C < 40 (mg/dl)				
No	14.5 (10.3–20.4)	9.7 (6.7–14.1)	1	1
Yes	16.0 (9.8–25.5)	10.8 (6.4–18.3)	1.12 (0.59–2.13)	1.87 (0.62–2.26)
Hypertension				
No	11.8 (7.9–17.3)	7.9 (5.2–12.0)	1	1
Yes	20.0 (13.4–29.2)	13.3 (8.6–20.7)	1.77 (0.96–3.24)	1.60 (0.86–2.96)
Mets				
No	13.2 (9.6–18.0)	8.7 (6.2–12.1)	1	1
Yes	33.3 (18.2–55.7)	28.8 (14.4–57.7)	3.22 (1.49–6.96)	3.55 (1.51–7.03)
Paternal obesity				
Non-obese	13.5 (9.3–19.5)	8.6 (5.8–12.8)	1	1
Obese	17.5 (7.7–37.0)	11.4 (4.7–27.4)	1.32 (0.50–3.47)	1.33 (0.51–3.49)
Maternal obesity				
Non-obese	9.7 (6.2–14.9)	5.9 (3.7–9.4)	1	1
Obese	30.0 (20.6–42.6)	23.6 (15.2–36.5)	3.85 (2.04–7.29)	4.43 (2.33–8.45)
Paternal educational level				
Diploma or lower than diploma	14.8 (10.5–20.7)	10.0 (6.9–14.4)	1	1
Higher than diploma	13.9 (6.0–20.2)	8.8 (3.6–21.0)	0.91 (0.35–2.35)	0.96 (0.37–2.51)
Maternal educational level				
Diploma or lower than diploma	14.2 (10.4–19.2)	9.3 (6.7–12.9)	1	1
Higher than diploma	22.2 (10.7–42.9)	18.6 (8.3–41.3)	1.81 (0.76–4.30)	1.82 (0.77–4.34)

Overweight, 1SD < BMI-for-age ≤ 2SD based on WHO criteria; *WC* waist circumference; *FPG* fasting plasma glucose, *HDL-C* high-density lipoprotein cholesterol, *TG* triglycerides; Paternal obesity, Father’s BMI ≥ 30 kg/m^2^; Maternal obesity, Mother’s BMI ≥ 30 kg/m^2^; Hypertension, SBP and/or DBP ≥90th percentile for gender and age; Mets, Metabolic syndrome for children based on the definition of Cook et al. work

^a^Adjusted for age at baseline

Table 5 presents the cumulative incidence and incidence density rate over the whole population, stratified by different age groups i.e. 7–9 (*N* = 559) and 10–11 (*N* = 474) years old; cumulative incidence was 22 and 10.8% in these age subgroups, respectively. Kaplan-Meier curve (Fig. 2b) also shows that children in the 7–9 year old group compared to their counterparts in 10–11 year old group are at increased risk of obesity (Log-rank test: 12.6, *P* <  0.001). Table 5 and Kaplan-Meier curves (Figs. 2c and d) are further stratified by gender and different age groups (7–9 and 10–11 years old) indicating that both boys and girls in 7–9 year age group are at greater risk of incidence of obesity in comparison to their 10–11 year old counterparts (Log-rank test: 10.91, P <  0.001 and Log-rank test: 2.65, *P* = 0.103, respectively). In cox regression models after adjustment for relevant confounders, age group 7–9 years had higher risk for development of obesity compared to age group 10–11 years. Corresponding adjusted HRs for whole population, boys and girls were 7.40 (CI 95%, 4.32–12.56), 11.76 (CI 95% 5.35–26.31) and 6.06 (CI 95% 2.69–13.69), respectively (reference category, age group 10–11 years).

**Table 5:** Cumulative incidence and incidence rate (in 1000 person year) stratified by gender and age groups

Age Group	7–9 years old (N = 559)			10–11 years old (N = 474)		
	Boys	Girls	Total	Boys	Girls	Total
Cumulative incidence	26.4 (20.2–34.1)	17.6 (12.5–24.5)	22.0 (17.8–27)	10.8 (6.4–17.9)	10.7 (6.5–17.4)	10.8 (7.5–15.3)
Incidence rate (in 1000 person year)	20.4 (15.0–27.7)	12,0 (8.2–17.3)	15.9 (12.5–20.1)	7.3 (4.2–12.6)	7.0 (4.1–11.8)	7.1 (4.9–10.4)

## Discussion

This longitudinal cohort study shows a relatively high incidence of childhood obesity (17%) after over 10 years of follow-up in an urban population of the Tehranian children, which was higher in boys than in girls. Younger non-obese children (7–9 years old) are at greater risk of obesity, compared to older non-obese children (10–11 years old), supported by a cumulative incidence of obesity equal to 22.0% vs 10.8% respectivley. Moreover, four parameters are associated with obesity incidence, including being overweight, having higher WC, childhood MetS and parental obesity.

Although many studies have reported the prevalence of childhood obesity before, there is paucity of data regarding incidence of obesity in childhood. Few studies have investigated this subject in developed countries. Cunningham et al. [10] reported a cumulative obesity incidence of 11.9% in the U.S children (aged 5–14) which was significantly lower than what we roported, they also showed the incidence of obesity is more likely to occur at a younger age, particulary among overweight 5-year-old children. Moreover, a study from United Kingdom (UK) carried out in a large contemporary cohort of English children, compared childhood obesity incidence in a subsample of children and reported the incidence of obesity to be 5.1, 6.7, 1.6% in early-childhood (3–7 years), mid-childhood (7–11 years) and late-childhood (11–15 years), respectively; with the highest peak in the mid-childhood age group [9]. Compared to results of these two studies we demonstrated higher incidence of childhood obesity (17%), which can be explained by differences in inclusion and exclusion criteria, study sample size, geographical location and population characteristics of the study samples. For example, exclusion of overweight children at the baseline in a cohort of English children might be a reason of this lower incidence reported for childhood obesity, whereas we only excluded obese children at baseline. Morover, both the above mentioned studies [9, 10] had larger sample sizes than this study (US: 7738 and UK: 4283 subjects vs. this study: 1033). Another reason for discrepancies between this study results and these two previously mentioned studies is employing different definition of childhood obesity; while we applied the definition of WHO, Cunningham et al. used Centers for Disease Control and Prevention (CDC) definitions and the UK’s study used The International Obesity Task Force(IOTF) definitions. As noted by Kelishadi et al., the definition of obesity (e.g., WHO, CDC, IOTF) may contribute to an over or under-estimation of obesity and make comparisons across studies difficult [8]. Other important factors are globalization and epidemiologic transition, currently occurring in Iran - a developing country. The key aspect of this epidemiologic transition is an increase in the incidence and prevalence of chronic non-communicable diseases (obesity, diabetes, hypertension and cardiovascular disease) [17]. This change is the consequent of the “nutritional transition”; which is occurring rapidly in Iran. This phenomenon is the result of overconsumption of simple sugars, saturated oil and processed food [18].

Findings of incidence studies can help health policy makers to focus implementation of preventative strategies on high risk subgroups. For reducing the burden of childhood obesity, results of current study could guide national program implementers to find best targets for anti-obesity interventions. Based on this study’s analysis [and in line with previously mentioned findings [9, 10]]; younger non-obese children (7–9 years old) have the highest risk for obesity development. The higher incidence of obesity at younger ages emphasizes the importance of prevention of obesity in the earlier years of childhood, a critical time to promote healthier eating behavior and life style that would prevent obesity [19] .

In agreement with Cunningham et al.’s findings, current study also demonstrated that boys had a relatively higher incidence of obesity than girls [10]. However, in our study while boys showed higher level of cumulative incidence in the 7–9 year age group, compared with girls, both genders had similar incidence for obesity in the 10–11 year old group. Moreover, in line with our results, a meta-analysis study reported higher trend in prevalence of obesity in boys than girls in Iran [8]. There are several possible explanations for this higher incidence of obesity in boys; it might be a reflection of changing body composition that occurs during puberty and is earlier and more continuous in girls, as well as some behavioral differences in the two genders [20, 21].

Regarding the prevalence and incidence of childhood obesity in TLGS population, recently, a study was carried out by Mottaghi et al., children aged 3–7 yr. at baseline were followed up for 10 years [22]. Using CDC’s definition of obesity, Mottaghi et al. reported a 18.8% cumulative incidence of obesity for normal weight children over 10 years of follow-up, which was much greater than what we observed for this group of children with same time of follow-up (7.7%). This discrepancy is probably because of age difference of study populations, while mean age of children entering their study was 5.3 years, in this study it was 9.2 years. In addition to Mottaghi et al’s results regarding incidence of obesity, we provided age subgroup analysis of obesity incidence allowing us to claim that age group itself is an important risk factor for obesity incidence, independent of any other variable including time.

Prevalence-based cross-sectional data in developing countries have addressed unhealthy nutrition, physical inactivity, socioeconomic status, area of residence, socio-cultural factors and genetic as risk factors associated with childhood obesity [23]. Other than age group and gender discussed earlier, this study reports childhood overweight, having higher WC, MetS and parental obesity as the best predicators of obesity incidence. It is already well known that childhood overweight increases the probability of incidence of obesity [24]; consistent to this, in current study a positive correlation of high childhood BMI and WC with obesity incidence was detected. As demonstrated earlier, there is a risk of an increased adverse cardiovascular outcomes and obesity in children with MetS [25], an association supported by this study’s findings, suggesting a 2.77 HR for obesity incidence in children with MetS.

There are numbers of strengths in this study. To the best of our knowledge, this study is the first population-based representative cohort which reports childhood obesity incidence and its associated demographic, anthropometric, metabolic, and socioeconomic risk factors in Iran and the middle east and north africa (MENA) region. The longitudinal design and having a relatively long follow-up period allowed us to assess the gender stratified incidence of obesity and its risk factors. Last but not least, is the use of direct measurements, instead of self-reported data for both children and parents.

We are also aware that our study has several limitations; first, our subjects were selected from TLGS, an urban-based population cohort in district 13 area of Tehran, with limited potential of generalization to the whole population of Iran, especially in rural areas. Second, taking account some variables and cofounders like dietary habits, socioeconomic status, physical activity, maternal smoking status during pregnancy, and psychological factors was beyond the scope of this study, even though they also could play a role in obesity incidence. Third, the drop out rate of 26.3%; importantly, our loss to follow-up participants had statistically significant higher baseline BMI and WC, suggesting that our results might even underestimate rates of obesity incidence in Tehranian children and adolescents.

## Conclusion

This study shows a significantly high childhood obesity incidence in Tehran, capital city of a developing country. To prevent incidence of obesity, we suggest earlier weight control plans in childhood, particularly before the age of 7. Moreover young children who suffer from overweight, WC above 90th percentile, MetS and parental obesity are the best targets for intervention against childhood obesity. However, further cohort studies with larger sample sizes and wider age group coverages are needed for better identification of high risk groups by exploring more risk factors involved in the development of obesity in children.

## Abbreviations

BMI: Body mass Index
FPG: Fasting plasma glucose
HDL-C: High -density lipoprotein cholesterol
HRs: Hazard ratios
MetS: Metabolic syndrome
TC: Total cholesterol
TG: Triglyceride
TLGS: Tehran Lipid and glucose Study
WC: Waist circumference
